# Dynamic restoration mechanism of plant community in the burned area of northeastern margin of Qinghai-Tibet Plateau

**DOI:** 10.3389/fpls.2024.1368814

**Published:** 2024-07-25

**Authors:** Zizhen Li, Jia Wei, Xiaolei Zhou, Qing Tian, Wanpeng He, Xueping Cao

**Affiliations:** ^1^ College of Forestry, Gansu Agricultural University, Lanzhou, China; ^2^ Research Institute of Forestry, Chinese Academy of Forestry, Beijing, China; ^3^ Gansu Academy of Agricultural Sciences, Lanzhou, China

**Keywords:** post-fire, plant characteristics, soil properties, community stability, restoration, partial least-squares path modeling

## Abstract

Forest fires play a pivotal role in influencing ecosystem evolution, exerting a profound impact on plant diversity and community stability. Understanding post-fire recovery strategies holds significant scientific importance for the ecological succession and restoration of forest ecosystems. This study utilized Partial Least Squares Path Modeling (PLS-PM) to investigate dynamic relationships among plant species diversity, phylogenetic diversity, soil properties, and community stability during various recovery stages (5-year, 15-year, and 23-year) following wildfires on the northeastern margin of the Qinghai-Tibet Plateau. The findings revealed: (1) Over time, species richness significantly decreased (*p*< 0.05 or *p*< 0.01), while species diversity and dominance increased, resulting in uniform species distribution. Community stability progressively improved, with increased species compositional similarity. (2) Throughout succession, phylogenetic diversity (PD) significantly decreased (*p*< 0.01), accompanied by rising Mean Pairwise Distance (MPD) and Mean Nearest Taxon Distance (MNTD). Net Relatedness Index (NRI) shifted from positive to negative, indicating an increasing aggregation and dominance of plants with similar evolutionary traits in burned areas. Early succession witnessed simultaneous environmental filtering and competitive exclusion, shifting predominantly to competitive exclusion in later stages. (3) PLS-PM revealed that in the early recovery stage, soil properties mainly affected community stability, while species diversity metamorphosed into the primary factor in the mid-to-late stages. In summary, this study showed that plant diversity and phylogenetic variation were successful in revealing changes in community structure during the succession process. Soil characteristics functioned as selective barriers for plant communities during succession, and community stability underwent a multi-faceted and dynamic process. The soil-plant dynamic feedback continuously enhanced soil conditions and community vegetation structure thereby augmenting stability. Post-fire vegetation gradually transitioned towards the original native state, demonstrating inherent ecological self-recovery capabilities in the absence of secondary disturbances.

## 1 Introduction

Wildfire, as a globally prevalent and significant ecological disturbance, has drawn wide attention to its impact on plant biodiversity and ecosystem functions (Brose and Hillebrand, 2016; Seidl et al., 2016). The occurrence of wildfires plays a crucial role in the functionality, structure, and dynamics of plant communities (Pausas et al., 2008; Shryock et al., 2015; Sagra et al., 2018) and is a key force driving ecosystem evolution (Bond and Keeley, 2005; Fernandes, 2013). Forest fires exhibit a dual nature: while low-intensity and periodic burning disturbances can enhance forest structure, promote nutrient cycling, and facilitate tree regeneration, growth, and development, high-intensity fires can disrupt forest structure and function, leading to ecosystem degradation or imbalance (Baker and Shinneman, 2004). Fire disturbance acts as a filter for plants with specific traits and regenerative strategies, serving as a key driver for the renewal and succession of plant community structure (Day et al., 2020). Ecological succession, including vegetation recovery, can strongly influence the post-fire ecosystem structure and function (Scheiner and Willig, 2008). Due to direct and indirect impacts on soil’s physical, chemical, and biological properties, vegetation recovery is a crucial process for ecosystem function restoration (Knelman et al., 2015). Monitoring the recovery of soil-plant interactions in stable and disturbed ecosystems is an effective approach to understanding the resilience of ecosystems (Certini, 2005; Doblas-Miranda et al., 2015). Therefore, a more in-depth study is needed to comprehend the impact of fire on vegetation succession, community renewal, and the long-term changes in community structure stability and soil properties during recovery. This will contribute to understanding the temporal and successional variations in post-fire plant diversity and community stability, providing effective management measures for ecological conservation at specific stages of forest succession.

Furthermore, studies on biodiversity changes during community succession or vegetation restoration have predominantly focused on species diversity, neglecting phylogenetic diversity (Zhang et al., 2004). Phylogenetic diversity can be used to infer the impact of historical evolutionary processes on existing communities (Webb et al., 2002) and is an important aspect of biodiversity (Flynn et al., 2011). Phylogenetic diversity is crucial for unraveling the mechanisms of community assembly and biodiversity changes in vegetation restoration (Li et al., 2018; Chen et al., 2021; Yue and Li, 2021; Li et al., 2023d). Utilizing phylogenetics to comprehend trait evolution in ecosystems experiencing wildfires can reveal patterns of evolutionary correlations among traits and the sequence of appearance of different fire-related traits (Schwilk and Ackerly, 2001; Pausas and Verdú, 2005); however, these studies have been constrained by the lack of molecular data for phylogenetic determination. In recent years, the abundant emergence of such data has enabled researchers to generate time-calibrated phylogenetic trees, making it possible to identify the origin and evolution of features associated with wildfires (Lamont and Downes, 2011; Lamont et al., 2011; Pausas and Schwilk, 2012). In this study, we use phylogenetics for the first time to interpret post-fire vegetation succession strategies.

In recent years, frequent local forest fires in the northeastern margin of the Qinghai-Tibet Plateau (QTP) have led to the destruction of forest ecosystems, changes in community structure, a reduction in biodiversity, and loss of ecological functions (Zhao et al., 2021; Zhou et al., 2022b). Post-intense fire disruption led to a reduction in soil total organic carbon, recalcitrant organic carbon, and particulate organic carbon content. Simultaneously, soil phosphatase and peroxidase activities increased, while urease activity decreased (Lu et al., 2022). The consumption of surface litter by fire results in a decrease in soil water-holding capacity (He et al., 2023). Additionally, the interspecific niche overlap among shrub species increases (Zhao, 2022). The niche overlap and interspecific competition among herbaceous plants initially increased and then decreased through different succession stages (Zhou et al., 2023). However, the existing understanding is fragmentary and incomplete, and the community succession process and mechanism of the whole system are still unknown. There is a lack of systematic research on the interactions and patterns of change between soil properties, plant diversity, and community stability at different stages of post-fire recovery. Moreover, research on the changes in species and phylogenetic diversity of plant communities in different recovery stages in burned area is scarce.

This study focuses on the northeastern margin of the Qinghai-Tibet Plateau to explore the dynamic recovery process of plant communities following fire disturbance. Using the PLS-PM method, we simulate vegetation changes over different recovery stages (5-year, 15-year, and 23-years intervals post-fire) and link relevant parameters to investigate the impact of soil properties, plant diversity, and phylogenetic diversity on community structure stability after forest fires. The study aims to address the following key questions: (1) What are the composition and diversity characteristics of plant communities at different stages of vegetation recovery? (2) How do the properties of the soil influence community vegetation at different recovery stages? (3) How do plant community structural characteristics, soil properties, and community stability trade off and respond to each other at different stages of restoration?

## 2 Materials and methods

### 2.1 Site description

**Figure 1:**
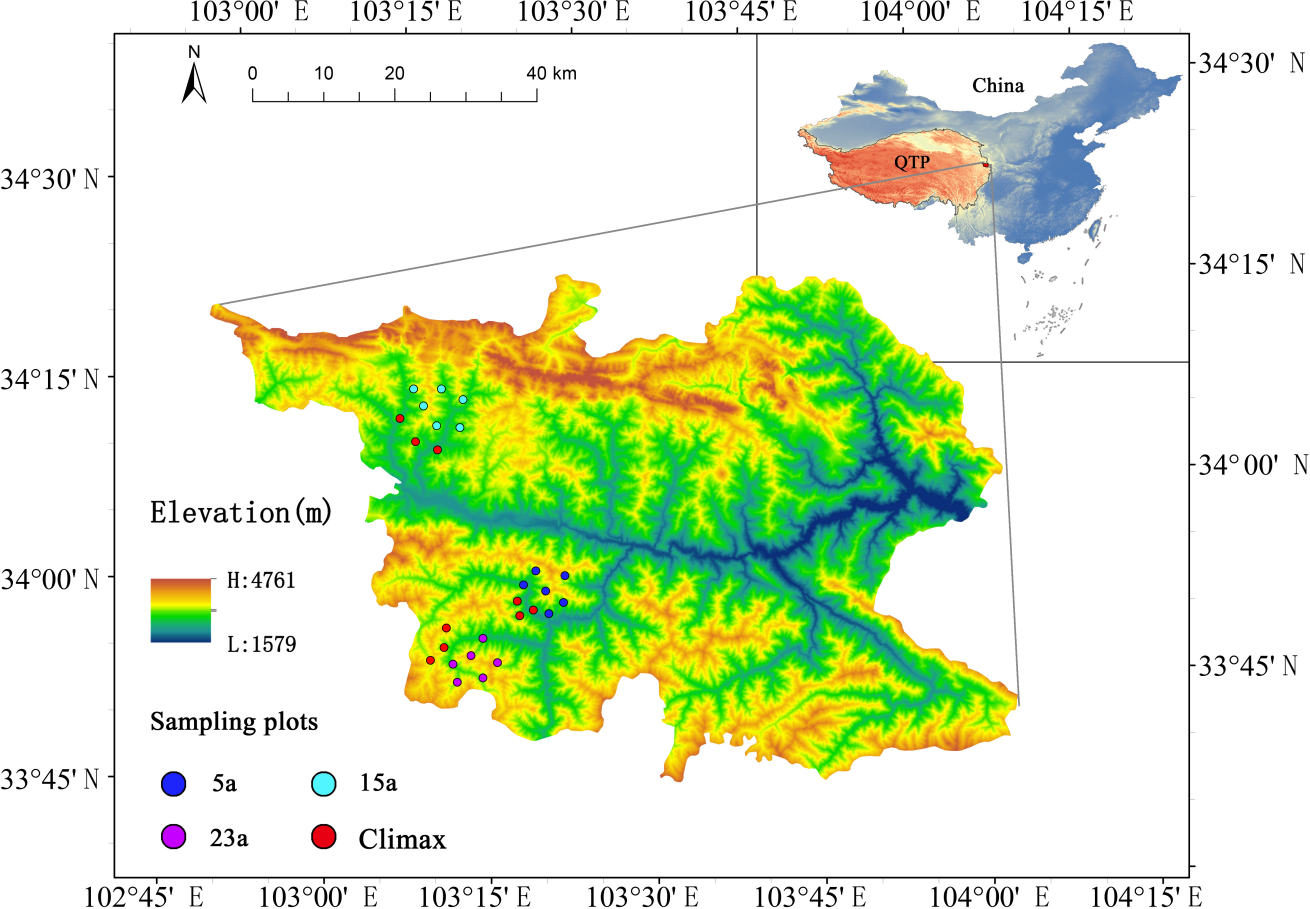
Spatial distribution of the study sites. 5a, 15a, 23a respectively represent the experimental sampling points of 5 years, 15 years and 23 years after the fire; Climax community represents the unburned control experimental plots in different recovery stages.

The northeastern margin of the Qinghai-Tibet Plateau, within the Bailongjiang Natural Reserve in Diebu County, Gansu Province, was chosen as the investigation site for this study. The sampling ranged from 33°49’18.84’’ N to 34°38’38.00’’ N and from 103°12’48.69’’ E to 103°21’22.14’’ E, and elevations ranging from 2575 to 3410 m (**Figure 1**). This area is a major watershed between the Yangtze River and Yellow River basins and is a part of the hilly vegetation zone of the Gannan Plateau (Zhao et al., 2021). It is characterized by a predominant transitional climate change between the North Subtropical Zone and the cold climate along the eastern boundary of the Qinghai-Tibet Plateau. The annual average temperature is 7.5°C, with an average annual precipitation of 568 mm, evaporation of 1444.2 mm, and 2308.0 hours of annual average sunshine. The annual average relative humidity ranges from 52% to 76% (based on data from 2000 to 2020 provided by the China Meteorological Data Service Center, http://data.cma.cn). The terrain has a relative elevation difference of about 1200 m and an average slope of 30° - 50°. The average soil depth is approximately 70 cm (Zhao et al., 2023), and the predominant soil types are mountain chestnut soil or mountain gray-brown soil (**Supplementary Table S1**). Due to the local topography, the climate exhibits significant vertical variation (Yang et al., 2023; Zhou et al., 2023). Based on the forest fire records from the local forestry bureau, the region has experienced frequent forest wildfires. The experimental design utilizes three areas severely burned in the summers of 1997, 2005, and 2015, representing natural recovery stages of 23 years (23a), 15 years (15a), and 5 years (5a), respectively (**Figure 1**). These areas demonstrate similarities in vegetation, soil, and climate to minimize variability from other factors. According to the fire scope provided by the local forestry bureau, these areas have not experienced repeated burning. Prior to the fires, the original plant community consisted mainly of a mixed forest of *Picea asperata-Abies fargesii*. Following the severe fires, these sampling sites were characterized by the total destruction of ground-level biota and the ecosystem at large, leaving only standing or fallen dead trees. Local authorities then promptly enacted enclosure measures to facilitate the natural regeneration of biota and the ecosystem at these locations.

### 2.2 Experimental design and data collection

According to our experimental design, field surveys and soil sampling were conducted during the peak period of plant community structure from July to August, 2021. A total of 6 plots, measuring 20m × 20m, were set up individually at burned locations 5a, 15a, and 23a post-fire, in addition to three control plots of 20m × 20m in nearby unburned forest regions individually. Additionally, elevation, slope, aspect, and geographic coordinates were recorded for each sample point (**Supplementary Table S1**). Employing the ‘five-point method’, five 5m × 5m shrub subplots were established in the east, south, west, north, and center of each large plot. This resulted in 30 subplots for each of the 5a, 15a, and 23a burned areas, and 45 subplots for the unburned forest area were established. For each subplot, shrub characteristics, including species, individual (or cluster) count, average height, base diameter, crown width, and crown coverage, were determined. To further assess herbaceous and soil components, an additional 1m×1m herb subplot was established within each shrub plot, recording herbaceous plant species, individual (or cluster) count, average height, and crown coverage. After investigating the characteristics of herbaceous, soil samples were collected at depths of 0-10cm, 10-20cm, and 20-40cm, according to the thickness of the soil layer above the rock layer. Samples from the same soil layer within each site were mixed to obtain composite soil samples, sealed in self-sealing bags, labeled, and stored. Subsequently, the soil samples were brought to the laboratory, air-dried, sieved through a 100-mesh sieve, and subjected.

#### 2.2.1 Soil analyses

Assessment of soil properties is important for understanding the dynamics of community structure and regeneration strategies at various post-wildfire recovery stages. The physicochemical properties in the soil, such as organic carbon, nitrogen, phosphorus, and potassium, are directly linked to plant growth and nutrient acquisition, serving as essential components for the normal physiological activities of plants (Moya et al., 2019). These elements play a crucial role in the regeneration and succession processes of plants (Raiesi and Pejman, 2021; Yao et al., 2021). Organic carbon serves as a carbon source (Pellegrini et al., 2022; Li et al., 2023a), while nitrogen, phosphorus, and potassium are key elements for plant growth and development (Yang et al., 2018). Changes in soil pH may trigger the succession and regeneration of plant communities, facilitating the replacement of less-adaptive plants by those with stronger adaptability (Certini, 2005; Lu et al., 2007). In this study, after digestion with 0.5 mol·L^-1^ potassium dichromate and sulfuric acid, the soil organic carbon (SOC) content was determined using the 0.3 mol·L^-1^ ferrous sulfate titration method. Soil pH was measured in a 1:5 soil-water suspension using a pH meter (Mettler-Toledo S220, Switzerland). Total nitrogen (TN) content was determined using the Kjeldahl method (Kjeldahl, 1883), using a Kjeldahl nitrogen analyzer (NKD-6200, YIHON Technologies, CN). Total phosphorus (TP) content was measured by the molybdenum antimony blue colorimetric method after digestion with sodium hydroxide. Total potassium (TK) content was determined using a flame photometer (BWB-XP, BWB Technologies, UK) after mixed digestion with concentrated sulfuric acid and perchloric acid. Ammonium nitrogen (NH_4_^+^-N) and nitrate nitrogen (NO_3_–N) were extracted with 2 M KCl_2_ and determined using a continuous segmented flow analyzer (SEAL-AA3, SEAL Analytical Limited, UK). Available phosphorus (AP) was determined by the molybdenum antimony blue colorimetric method after extraction with 0.5 mol·L^-1^ sodium bicarbonate for neutral and alkaline soils, and 0.03 mol·L^-1^ ammonium fluoride and 0.025 mol·L^-1^ hydrochloric acid for acidic soils. Available potassium (AK) was extracted with 1 mol·L^-1^ ammonium acetate (NH_4_OAc) and determined using a flame spectrophotometer (BWB-XP, BWB Technologies, UK).

#### 2.2.2 Species diversity and phylogenetic diversity

Studies on species diversity are crucial for understanding plant regeneration and succession, playing a vital role in revealing the stability of ecosystems, ecological niche utilization, dynamics of community structure, resistance, resilience, and ecological succession models (Li et al., 2023b). This research provides a comprehensive perspective on the development of plant communities. Phylogenetic diversity, by measuring species distribution on the phylogenetic tree, average phylogenetic distance, average phylogenetic heterogeneity, as well as relative abundance and adaptability, offers deep insights, revealing ecological niche utilization, competitive dynamics, and changes in community structure during the process of plant community succession (Jiao et al., 2021). The integrated analysis of these research components significantly guides the unveiling of strategies for community regeneration, succession, as well as in maintaining community stability (Jiang et al., 2022).

According to the quadrat records, species diversity indexes, including the Margalef index, Shannon-Wiener index, Pielou index, and Simpson index, were calculated using the methods described in Ma et al. (1995) within R v4.2.3 (R Core Team, 2020) using the ‘vegan’ package (Oksanen et al., 2013). To create species-level phylogenetic trees, we utilized the R package ‘V.PhylloMakert2’, based on the evolutionary tree of Jin and Qian (2022) (**Supplementary Figure S1**). Phylogenetic Diversity (PD) is the cumulative sum of all phylogenetic branch lengths on the phylogenetic tree, representing the complexity and conservation value of a community. In other words, a larger PD value indicates greater species complexity and higher conservation value in the community (Faith, 1992; Faith et al., 2004). Faith’s PD values were computed using the ‘pd’ function from the ‘picante’ package (Kembel et al., 2010). Furthermore, the picante package was employed to calculate the phylogenetic β-diversity between sample sites, measured as both the mean pairwise distance (MPD) and the mean nearest taxon distance (MNTD). Notably, MPD reflects the degree of aggregation of different taxa near the root node of the phylogenetic tree, whereas MNTD reflects the degree of aggregation near the terminal branch (Webb et al., 2008). Larger values of MPD and MNTD indicate more distant phylogenetic relationships within the community, suggesting a higher likelihood of competitive exclusion dominating species coexistence. Conversely, smaller values of MPD and MNTD imply closer phylogenetic relationships within the community, indicating a higher likelihood of habitat filtering dominating species coexistence (Webb et al., 2002; Miller et al., 2017). The Faith’s PD (Equation 1), MPD (Equation 2) and MNTD (Equation 3) were computed as follows:


(1) $$\text{PD}=\sum \left(d_{i}\right)$$

(2) $$\text{MPD}=\frac{1}{2}\left(\sum_{i=1}^{n_{a}}f_{i}\bar{d_{ib}}+\sum_{j=1}^{n_{b}}f_{j}\bar{d_{jb}}\right)$$

(3) $$\text{MNTD}=\frac{1}{2}\left(\sum_{i=1}^{n_{a}}f_{i}mind_{ib}+\sum_{j=1}^{n_{b}}f_{j}mind_{ja}\right)$$

Here, *d_i_* represents the branch length for species *i* on the phylogenetic tree, and *d_ib_*/*d_jb_* denotes the MPD between species *i*/*j* from sample *a* and all species from sample *b*. Variables *n_a_*/*n_b_* and *f_i_*/*f_j_* respectively stand for the number of species and the relative abundance of species *i*/*j* in sample *a/b*, while *mind_ib_*/*mind_jb_* represents the MNTD between species *i*/*j* in sample *a* and all species in sample *b*.

To analyze the community phylogenetic structure (clustering or overdispersion) and investigate potential ecological and evolutionary processes within communities, we calculated the net relatedness index (NRI) and net nearest taxon index (NTI) (Webb et al., 2002) using the ‘picante’ package. NRI is based on the MPD, providing an estimate of the average phylogenetic relatedness between all possible pairs of taxa within a sample. NRI primarily reflects the structure in the deeper parts of a phylogeny. On the other hand, NTI is based on the MNTD, estimating the mean phylogenetic relatedness between each pair of taxa in a sample to its nearest relative in the phylogeny. NTI reflects the structure in shallower parts of a phylogeny (Webb et al., 2002; Miller et al., 2017). At the community level, larger values of NRI and NTI indicate a more clustered phylogenetic structure, while smaller values suggest a tendency toward dispersion. When NRI and NTI approach 0, the phylogenetic structure of the community system tends to be more random (Du et al., 2022). The NRI (Equation 4) and NTI (Equation 5) values were computed as follows:


(4) $$\text{NRI}=-1\times \frac{MPD_{observed}-MPD_{random}}{\text{s}.\text{d}.\left(MPD_{random}\right)}$$

(5) $$\text{NTI}=-1\times \frac{MNTD_{observed}-MNTD_{random}}{\text{s}.\text{d}.\left(MNTD_{random}\right)}$$

#### 2.2.3 Community stability

Plant community stability was assessed using the inverse of the coefficient of variation (ICV) (Wu et al., 2014; Yang et al., 2017; Jiang et al., 2022). The ICV formula (Equation 6) as follows:


(6) $$\text{ICV}=\frac{\mu }{\sigma }$$

ICV represents the ratio of the average density (*μ)* of each species in the quadrat to the standard deviation (*σ)* of their respective densities. A greater ICV value indicates heightened community stability and reduced variability in species density.

### 2.3 Statistical analyses

Prior to the analysis, all variables underwent suitable transformations to meet normality criteria and ensure homogeneity of variances. Additionally, we evaluated the impact of restoration time (5a, 15a, 23a, and climax community) and soil depth (0 - 10 cm, 10 - 20 cm, and 20 - 40 cm) on soil properties using two-way ANOVAs with Duncan multiple comparisons. For investigating significant differences in plant diversity among different restoration years, a one-way ANOVA with Duncan multiple comparisons was employed. Nonlinear regression was used to better understand the relationship between community stability and plant diversity (species diversity, phylogenetic diversity) in different restoration years. To assess the relationships between species diversity and phylogenetic diversity, Pearson correlation analysis was employed. Furthermore, the significant effects of soil properties and plant diversity were analyzed using the Mantel test with package ‘ggcor’ (Oksanen et al., 2013).

### 2.4 Structure equation modeling: PLS-PM

Partial Least Squares Path Modeling (PLS-PM) analysis can overcome the limitations of multivariate normality and accommodate large sample sizes commonly encountered in linear structural relationship analyses. This method, a form of structural equation model, is capable of uncovering and predicting the relationships between observed and latent variables, as well as revealing and predicting interrelations among diverse latent constructs (Wold, 1966, 1980; Hair et al., 2021; Chen et al., 2022). PLS-PM allows for analyses even with a limited number of samples. The initial run of PLS-PM divided all measurement indicators into four latent variables: soil properties (e.g., TN, TP, TK, AP, AK, NH_4_^+^-N, NO_3_^–^N, SOC, pH); phylogenetic diversity (e.g., PD, MPD, MNTD, NRI, NTI); species diversity (e.g., Margalef, Shannon, Pielou, and Simpson); community stability (e.g., ICV). In order to augment the model’s goodness of fit and attaining an elevated level of confidence, the non-significant observed variables (factor load less than 0.7) and non-significant paths are eliminated. A reflection indicator loading greater than 0.7 was considered appropriate for the corresponding latent variables (Sanchez, 2013). The coefficient of determination (R^2^) represents the extent to which variance in the dependent latent variable is explained by its independent latent variables, and a higher R^2^ value indicates a more robust model (Cepeda-Carrión and Roldán-Salgueiro, 2005). The goodness-of-fit (GOF) index was used to evaluate the model quality (Sanchez, 2013). The PLS-PM analysis was conducted using the ‘plspm’ package in R 4.2.3.

## 3 Results

### 3.1 Characteristics of plant communities at various stages

**Figure 2:**
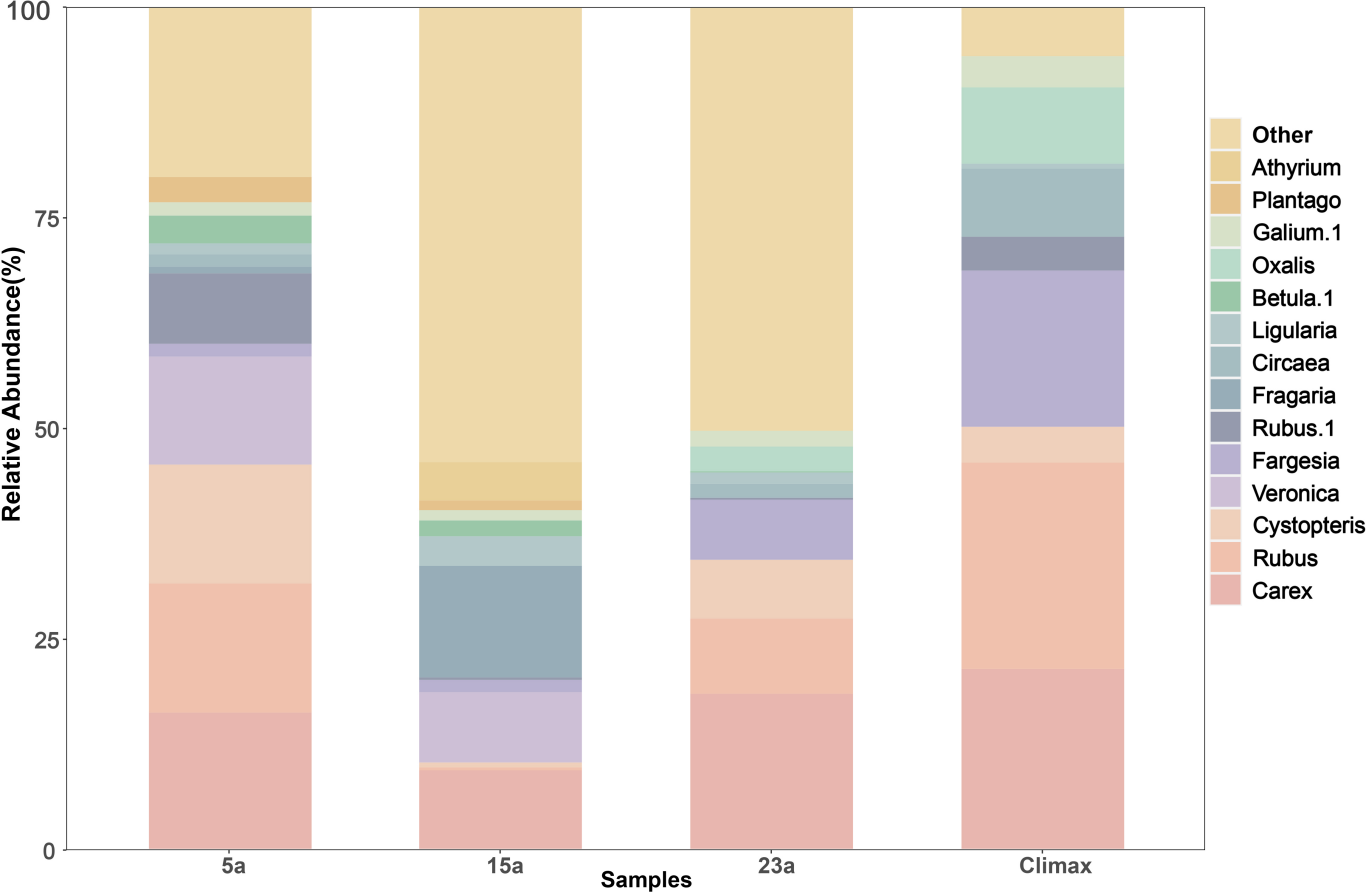
Relative abundance of plant genus-level in different recovery periods. Top 15 most abundant genera in this cohort.

The study plot contained a total of 174 plant species, belonging to 49 families and 108 genera (**Supplementary Table S2**). 5a years post-fire, the burned area harbored 90 species across 29 families and 57 genera, with a significant representation from the Asteraceae (26.7%). 15a years post-fire, the area included 71 species from 18 families and 44 genera, with primarily dominated by the Rubiaceae (7.0%), Polygonaceae (7.0%), Pinaceae (5.6%). 23a years post-fire, the region supported 70 species from 17 families and 39 genera, with a major concentration in the Asteraceae (34.3%) and Ranunculaceae (24.3%). The climax community comprised 105 species from 34 families and 69 genera, predominantly found in the Rosaceae (14.3%), Caprifoliaceae (9.5%), Apiaceae (7.6%). The main plants in the sampling plots were concentrated in *Carex, Rubus, Cystopteris, Fargesia, Ligularia*, etc. (**Figure 2**). The relative abundance of each genus species varied with restoration years. After 5 years of restoration, the dominant genus was Rubus (23.7%), represented by Rubus pileatus and Rubus amabilis. After 15 years of restoration, the dominant genus was transformed into Fragaria (13.2%). At the 23a-stage, Carex was the most abundant (18.4%) (**Supplementary Table S4**).

Across the four recovery stages, 24 species were consistently present, including *Actaea cimicifuga*, *Anaphalis sinica*, *Aster ageratoides*, *Berberis kansuensis*, *etc.* (**Supplementary Figure S2**, **Supplementary Table S3**). The consistent presence of these species across all stages could indicate their role as indicator species for monitoring ecological health or recovery in fire-affected areas. The resilience or adaptability of these species provides insights into ecological stability and succession dynamics. The total number of endemic plants peaked at the 5a stage and exhibited a gradual decline at the 15a and 23a stages. The 5a-stage boasted the highest number of endemic species, with 22 endemic species such as *Ajania potaninii, Caltha palustris, Clematis montana*. This was followed by the 15a-stage, which featured 21 endemic species including *Agrostis hugoniana, Athyrium sinense, Cotoneaster acuminatus, etc*. The climax community contained 19 endemic species, including *Aconitum tanguticum, Adoxa moschatellina, Allium victorialis, etc*. Additionally, the 23a-stage recorded 14 endemic species such as *Aconitum henryi, Anaphalis nepalensis, Chenopodium album*, *etc.*

### 3.2 Changes of plant diversity with recovery time

**Figure 3:**
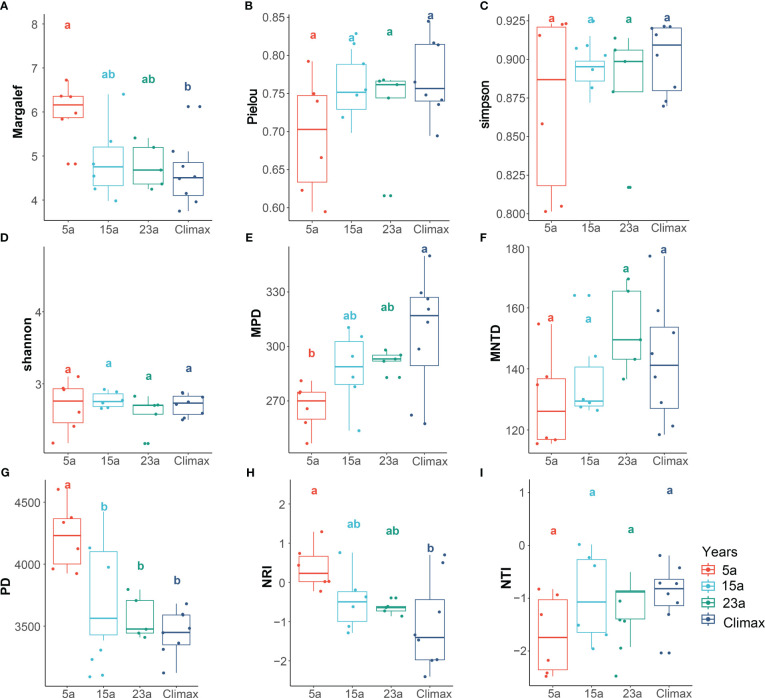
Plant community characteristics among the different recovery periods. Different lowercase letters (a, b, and ab) indicate significant differences (*p*<0.05) within each variable among the different restoration periods. Diverse colors signify distinct stages of recovery. Species diversity **(A–D)** includes Margalef, Pielou, Simpson, and Shannon; Phylogenetic diversity **(E–I)** includes PD, MPD, MNTD, NRI, and NTI.

Species diversity indices, including Margalef and Shannon, exhibited a decrease with increasing restoration duration across all sampled communities, with the Margalef index displaying significant variation in this trend (*p*< 0.05). Species diversity indexes show that most sharp increases or decreases occur between 5a and 15a stages, with the trend becoming more gradual over time. Regarding phylogenetic diversity, PD and NRI values across all sampled locations significantly decline with restoration duration (*p*< 0.001, *p*< 0.05, respectively). while MPD shows a significantly increase with recovery time (*p*< 0.05) (**Figure 3**, **Supplementary Table S5**).

**Figure 4:**
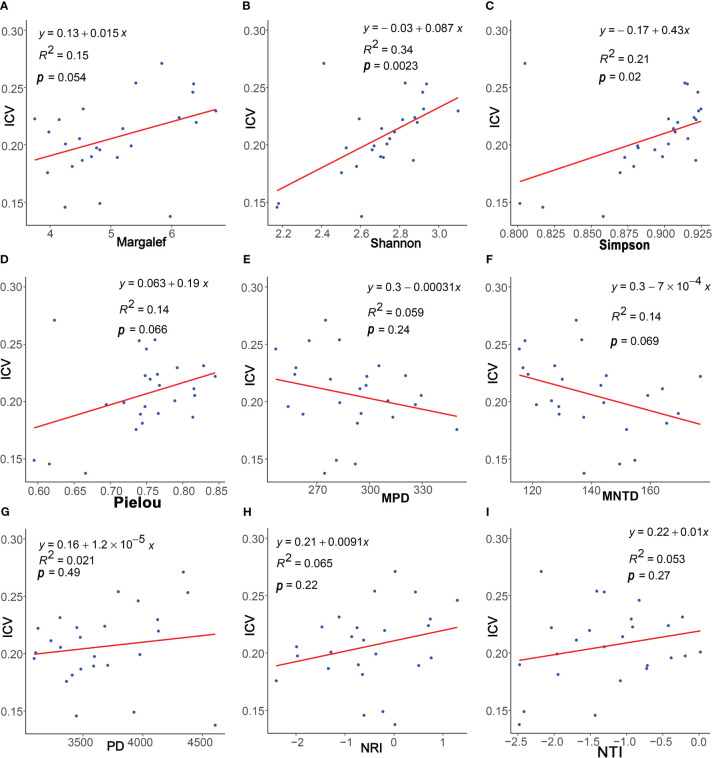
Linear regressions of plants characteristics and community stability. The plants characteristics represented by **(A–I)** are Margalef, Pielou, Simpson, Shannon, PD, MPD, MNTD, NRI, and NTI, respectively.

The results of the regression analysis (**Figure 4**) demonstrated a positive correlation between community stability and the four species diversity indices: Margalef index, Simpson index, Shannon index, and Pielou index. Particularly, significant correlations were observed with the Shannon (*p*< 0.01) and Simpson indices (*p*< 0.05). Regarding phylogenetic diversity, community stability exhibited non-significant positive correlations with PD, NRI, and NTI indices (*p* > 0.05) and non-significant negative correlation with MPD and MNTD indices (*p* > 0.05).

### 3.3 Response of soil physiochemical properties to community restoration

**Figure 5:**
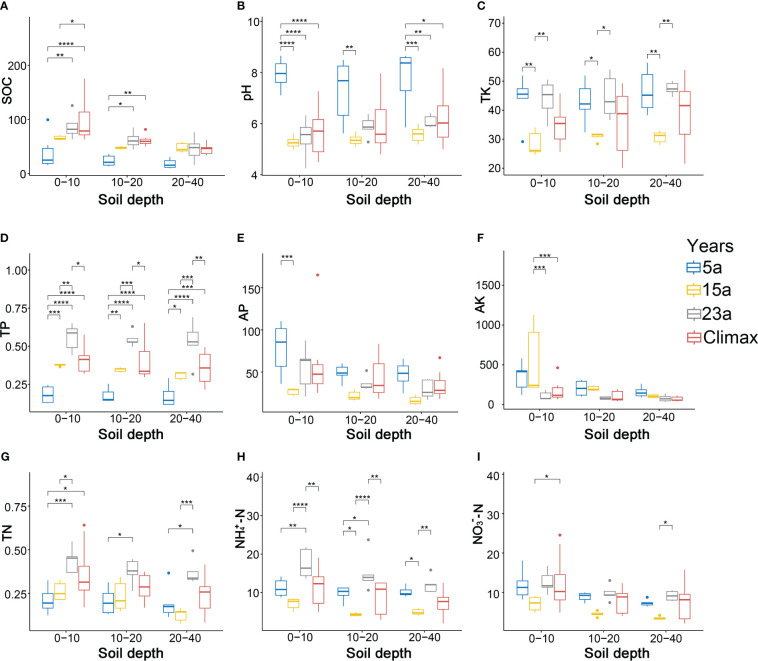
Soil properties among the different periods. Statistics for the correlation analysis are also indicated (* *p*< 0.05; ** *p*< 0.01; *** *p*< 0.001; and **** *p*<0.0001, respectively). Diverse colors signify distinct stages of recovery. **(A–I)** represent changes in SOC, pH, TK, TP, AP, AK, TN, $\text{N}{\text{H}}_{4^{+}}–\text{N}$ , and $\text{N}{\text{O}}_{3^{-}}–\text{N}$ at different stages of recovery and in different soil layers, respectively.

Soil physiochemical properties differed significantly among the different vegetation restoration periods (**Figure 5**, **Supplementary Table S6**). The SOC content in the same soil layer gradually increases with the progress of recovery time, and the effect of the fire on SOC decreases as the soil layer deepens (**Figure 5A**). The pH value in each soil layer is alkaline during the 5a recovery stage but acidic in the middle and late stages of restoration (**Figure 5B**). TK content is significantly higher in the 5a and 23a of restoration than in the 15a (*p*< 0.05, *p*< 0.01) (**Figure 5C**). TP content varies significantly (*p*< 0.01, *p*< 0.001, *p*< 0.0001) regardless of the various recovery stages and soil layers (**Figure 5D**). Additionally, TP content is significantly higher in the 23a-stage than in the climax community (*p*< 0.05, *p*< 0.01). As recovery progressed, the AP and AK contents in each soil layer gradually decreased, with significant changes observed only in the 0-10cm soil layer (*p*< 0.001) (**Figures 5E, F**). The significant increase in TN content is concentrated in the 0-10cm soil layer across the recovery period (*p*< 0.05, *p*< 0.001) (**Figure 5G**). In addition, as recovery progressed, the contents of TK, $\text{N}{\text{H}}_{4^{+}}–\text{N}$ , and $\text{N}{\text{O}}_{3^{-}}–\text{N}$ initially decreased and then increased (**Figures 5H, I**). The trend of $\text{N}{\text{H}}_{4^{+}}–\text{N}$ , content decreasing and increasing with recovery time is significant (*p*< 0.05, *p*< 0.01, *p*< 0.0001), while a similar trend in $\text{N}{\text{O}}_{3^{-}}–\text{N}$ content is not significant.

**Figure 6:**
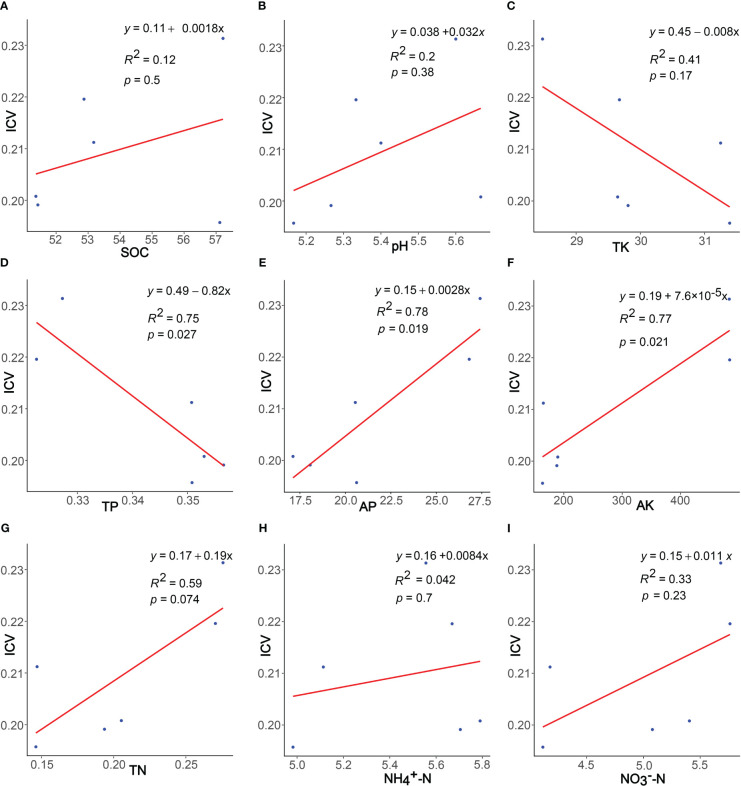
Linear regressions of soil properties and community stability. The soil properties represented by **(A–I)** are SOC, pH, TK, TP, AP, AK, TN, $\text{N}{\text{H}}_{4^{+}}–\text{N}$ and $\text{N}{\text{O}}_{3^{-}}–\text{N}$ , respectively.

Regression analysis results (**Figure 6**) indicate that community stability positively correlates with SOC, pH, AP, AK, TN, $\text{N}{\text{H}}_{4^{+}}–\text{N}$ , and $\text{N}{\text{O}}_{3^{-}}–\text{N}$ , while negatively correlating with TK and TP. Significantly, TP, AP, AK, and TN all show significant correlations (*p*< 0.05).

### 3.4 Changes of plant community stability

**Figure 7:**
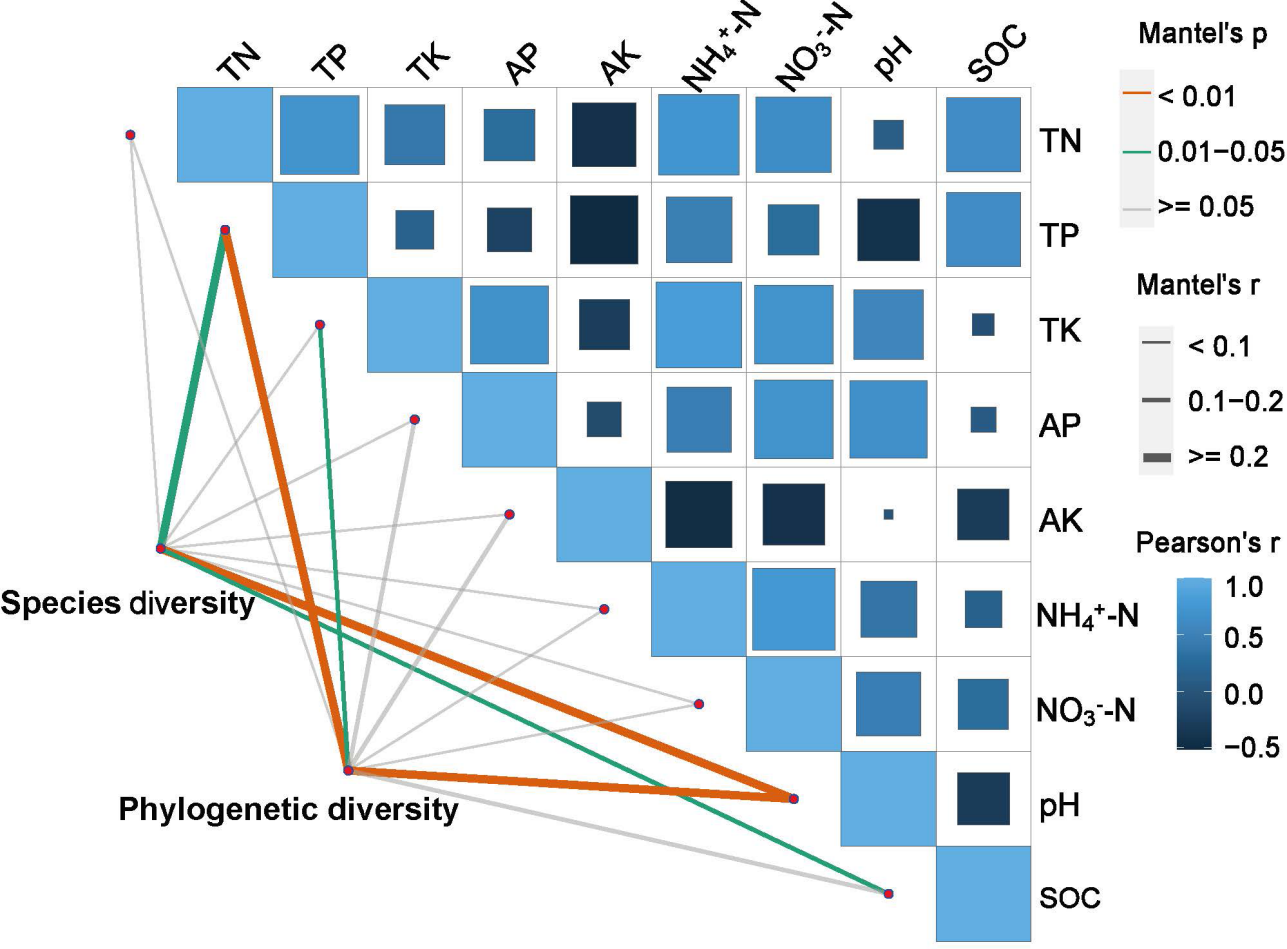
The mantel test on the relationship between plant diversity and soil properties. Legend: 0 represents no correlation, 1 represents the greatest correlation, and -1 represents the smallest correlation. The proportion of the square filled area represents the absolute value of the correlation. The value of the correlation is indicated by the shaded color and saturation.

The results of the Mantel test indicate that (**Figure 7**, **Supplementary Table S7**), when utilizing the criteria of R^2^ ≥ 0.2 and *p* < 0.05, a statistically significant relationship is discernible between plant diversity and soil chemical stoichiometry across various periods of restoration. Species diversity demonstrated a positive, significant correlation with TP, pH, and SOC (*p*< 0.05, *p*< 0.01, *p*< 0.05, respectively). Additionally, phylogenetic diversity exhibited a positive significant correlation with TP, TK, and pH (*p*< 0.01, *p*< 0.05, *p*< 0.01, respectively).

**Figure 8:**
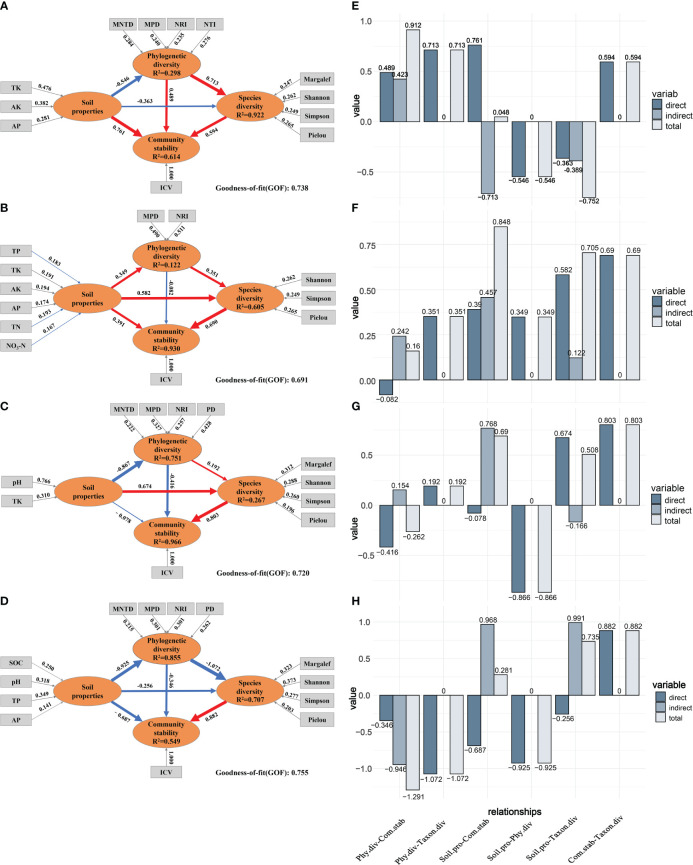
Partial least-squares path model (PLS-PM) of succession stages 5a **(A)**, 15a **(B)**, 23a **(C)**, and climax community **(D)**. Circles and rectangles indicate latent and manifest variables, respectively. Unidirectional cause-total-effect between latent variables is shown as an arrow with a path coefficient (red and blue arrows indicate positive and negative effects, and the thickness of the arrow line represents the size of the path coefficient in the model, respectively). The value above the arrow in the outer model represents the weight of the manifest variable. The R^2^ in a circle indicates the determination coefficient of each variable in the inner model. GOF > 0.600: indicates that the model has a good fitting degree. Direct, indirect, and total effect between variables in the inner model of different factors of 5a **(E)**, 15a **(F)**, 23a **(G)**, and climax community **(H)**. Phy.div, Taxon.div, Soil.pro, Com.stab correspondingly denote the abbreviations for phylogenetic diversity, species diversity, soil properties, and community stability.

To comprehensively explore the intricate network of relationships among soil properties, species diversity, phylogenetic diversity, and community stability across different post-fire recovery periods, PLS-PM was constructed (**Figure 8**). The results showed that soil properties had a significant direct positive effect on community stability at the 5a recovery stage (direct effect value, DEV: 0.761). However, as recovery time extended, this direct effect gradually diminished and turned negative by the 23a stage (DEV: -0.078). Despite the declining direct effect of soil properties on community stability, their indirect effects increased (indirect effect values, IEV: -0.713, 0.457, and 0.768). The direct effect of phylogenetic diversity on community stability was positive at 5a (DEV: 0.489), turned negative at 15a (DEV: -0.082), and became more negative at 23a (DEV: -0.416). Unlike soil properties and phylogenetic diversity, species diversity always had a positive direct effect on community stability across different recovery stages, with the magnitude of this effect increasing (DEV: 0.594, 0.690, and 0.803). The direct effect of soil properties on species diversity transitioned from negative at the 5a stage (DEV: -0.363) to positive at the 15a stage (DEV: 0.582), reaching its peak at the 23a stage (DEV: 0.674). While phylogenetic diversity initially had a strong positive direct effect on species diversity, this effect diminished as the recovery period progressed (DEV: 0.713, 0.351, and 0.192). Compared with the climax community (**Figure 8D**), soil properties, phylogenetic diversity and species diversity gradually shifted toward resembling those of the primary community during succession.

The 5a post-fire recovery stage (**Figures 8A, E**) indicated that soil properties, phylogenetic diversity, and species diversity all had positive direct effects on community stability. Notably, soil properties had a significant positive effect on species diversity and phylogenetic diversity (DEV: 0.594, 0.489). While soil properties generally had a positive impact, specific conditions at the 5a stage revealed negative direct effects on both phylogenetic diversity and species diversity (DEV: -0.546, -0.363). Phylogenetic diversity positively influenced community stability through direct and indirect effects. In contrast, soil properties had negative direct and indirect effects on species diversity. In the 15a stage (**Figures 8B, F**), among all direct effects on community stability, species diversity exerted the strongest positive effect, followed by soil properties, while phylogenetic diversity had a negative effect. Although phylogenetic diversity had a negative direct effect on community stability, its indirect effect was positive. The 23a stage (**Figures 8C, G**) showed that among the direct effect on community stability, only species diversity had a positive impact, while both soil properties and phylogenetic diversity had negative effects. Although the direct effect of soil properties on community stability was negative, its indirect effect was positive (IEV: 0.768). In the climax community, species diversity was the key factor influencing community stability (DEV: 0.882), while both soil properties and phylogenetic diversity exhibit negative impacts on community stability (DEV: -0.687, -0.346). The indirect effect of soil continues to have a significantly positive impact on community stability (IEV: 0.968).

## 4 Discussion

This study ascertained, for the first time, the post-fire recovery, and successional mechanisms on the northeastern margin of the Qinghai-Tibet Plateau, exploring the mutual interactions among plant community characteristics, soil properties, and community stability during different successional stages. Understanding these impacts is crucial for predicting forest ecosystem responses to post-fire conditions and comprehending the renewal strategies and recovery patterns of community species. This knowledge contributes to prioritizing adaptive management strategies to sustain ecosystem functions and services, enhancing fire resilience in vulnerable landscapes of the Qinghai-Tibet Plateau.

### 4.1 Restoration mechanism of plant community after fire

Species diversity serves as a reflection of the complexity and stability of community dynamics, species composition, forest structure, and community functionality (Harrison, 1999; Joyce, 2014; Moges et al., 2017). The recovery of plant community species diversity often serves as a crucial indicator for the restoration of degraded ecosystems (Winikoff et al., 2020). In the early stages of restoration, exposed surfaces, abundant sunlight, and nutrient-poor soils may be the primary drivers of plant community succession. Numerous herbs, such as *Carex crebra*, *Ligularia botryodes*, *Veronica polita*, *Saussurea amara* et al, are characterized by ease of propagation, photophilic tendencies, and tolerance to nutrient deficiency. These plants rapidly invade the burned areas, continuously establishing and reproducing. Utilizing their unique ecological adaptive properties, these plants become pioneer populations and even dominant populations in the community.

As succession progresses, the increase in shrub and tree seedlings occupies more living space and resources, leading to a reduction in herbaceous plants. This trend results in higher community dominance and diversity, with even distribution of species (**Figures 3**, **4**; **Supplementary Figure S2**). Previous research suggested that this phenomenon might be attributed to the continuous competition and utilization of spatial resources among species within and between burned areas, leading to a trend towards uniform distribution and higher community stability (Zhao et al., 2023). However, diversity indices for species classification may pose some limitations in assessing community stability, since evaluating them as mere fundamental research units could be difficult to discern the contributions of different species to system stability, ultimately affecting the judgment of species diversity on ecosystem stability (Hobbie, 1992; Wang et al., 2022).

The present study is unique as it not only examines the succession of vegetation quantity and species but also attempts to apply phylogenetics to explore the recovery of community structure. Phylogenetic diversity, often regarded as a crucial indicator reflecting functional diversity within ecosystems (Arthur et al., 2017), quantifies the evolutionary diversity of species, including phylogenetic relationship information among different species. It can effectively explain and predict the driving processes of ecosystems (Crisp et al., 2009), contributing to a deeper understanding of community succession strategies following forest fires. The core principles of phylogenetic diversity are guided by the theories of niche and neutral processes from ecological and genetic perspectives (Fisher and Mehta, 2014). It is based on phylogenetics and genetics, determining the contribution of a species to the diversity of a group. The former emphasizes deterministic processes such as habitat filtering and competitive exclusion as key drivers of community clustering, while the latter contends that stochastic processes, such as the diffusion of reproductive bodies, predominantly shape community clustering (Hubbell, 2001; Tilman, 2020, 17). For studies related to terrestrial ecosystem conservation and community stability, phylogenetic diversity surpasses species diversity indicators (Cadotte et al., 2011; Naeem et al., 2012; Jiao et al., 2021).

Our research indicates that, as the number of species decreased, the variation in phylogenetic diversity also decreased, suggesting that, in burned areas, plants with closer phylogenetic relationships evolve within the community. Plants with similar evolutionary characteristics gradually become dominant in the community after adapting to the habitat disturbed by fire (Faith, 1992; Faith et al., 2004; González-Orozco et al., 2016) (**Figure 3**), or, in other words, plants with functional similarities gradually become the main building species in the community, which is also the result of environmental filtering and selection (Shryock et al., 2015). The MPD and MNTD indices increased with recovery time, indicating that competitive exclusion is more likely to drive species coexistence within the entire burned area community succession process (Webb, 2000; Webb et al., 2002). The community undergoes loss and reconstruction of species richness and reorganization of phylogenetic relationships and functional distribution among species (Petchey and Gaston, 2002; Liu et al., 2022). In the 5-year stage, NRI and NTI indices produce inconsistent results (**Figure 3**). This may be due to the combined effects of habitat filtering and competitive exclusion, indicating that species turnover within the community was intense during this stage, showing both a trend of phylogenetic clustering and dispersion (Chen et al., 2021). In the middle to late stages of succession, the phylogenetic structure of the community showed a trend of dispersion, showing that competitive exclusion gradually becomes more prominent in clustering the community (Webb, 2000; Webb et al., 2002). Additionally, the filtering effect of the environment decreased over time, which was supported by the direct effect of soil characteristics on community stability (**Figure 8**). As vegetation continues to recover, some pioneer species face competitive exclusion from closely related species in the later stages of succession, leading to enhanced niche differentiation between species and resulting in a dispersed phylogenetic structure (Hao et al., 2019). Previous studies mentioned that processes such as habitat filtering and competitive exclusion may play an important role in vegetation recovery during different recovery stages after wildfires (Zhao et al., 2021; Zhou et al., 2023). Our evidence based on phylogenetic diversity precisely corroborates this assertion. The habitat filtering method based on environmental parameters, however, was not covered in the present study and needs further thorough investigation.

In addition, we observed that while community structure has essentially returned to pre-fire conditions in the late stages of recovery, differences in community vegetation were evident when assessed through phylogenetic diversity. This phenomenon may be attributed to ecological compensation effects (Zhao et al., 2022), wherein certain species exhibit stronger adaptability to post-fire conditions during the successional process, leading to the redistribution of ecological niches and changes in interactions among plants. Reduction or extinction of species in a forest ecosystem can result in an increase in other species, and dynamic compensation serves as a mechanism to maintain overall species diversity and community stability (McCann, 2000; Zhao et al., 2022).

The phylogenetic diversity and ecosystem stability of vegetation communities after the fire showed a positive correlation. This is primarily attributed to two reasons. Firstly, the ecological differentiation values of dominant species were positively correlated with the differences in the evolution and genetic material of species. The diversity in ecological differentiation can effectively reduce competitive interactions for resource utilization, resulting in a more productive ecosystem with a greater variety of species across different trophic levels, enhancing the stable coexistence of species in the ecosystem (Song et al., 2018). Secondly, due to the conservatism of species evolution, species with greater phylogenetic distances exhibit greater trait differences (Li and Yue, 2020). The greater functional differences among species in the ecosystem, along with their distant phylogenetic relationships, led to higher efficiency in the effective utilization of nutrients in the ecosystem, thus elevating its functional and ecological stability (Wang et al., 2022).

### 4.2 Post-fire soil stoichiometric characteristics

Nutrient-rich soils play a crucial role in plant growth, development, and the shaping of plant community composition and diversity (Xing and He, 2019). The recovery of soils after a fire is essential for maintaining its functionality including nutrient cycling, biochemical degradation, and sustainable plant growth, ensuring the long-term health and sustainability of ecosystems (Raiesi and Pejman, 2021). As succession progressed, TN and TP gradually increased, consistent with previous research findings (Segura et al., 2016). This trend may be attributed to the recovery of vegetation over time, increased input of litter during plant community succession, and microbial decomposition of root residues and the litter layer, enhancing soil nitrogen and phosphorus content, with a higher impact on nitrogen and phosphorus enrichment levels (Mazzoncini et al., 2016). Additionally, the accumulation of soil organic matter provides a rich nutritional substrate for microbes, enhancing microbial activity and accelerating the accumulation of soil N and P elements (Zhou et al., 2022a).Interestingly, during the 23-year stage, the TP and TN content in all soil layers was higher than that in the unburned area (**Figure 5**). This could be attributed to changes in vegetation composition and structure during the later stages of community succession, resulting in alterations in plant species and quantities. Certain plants may possess higher nutrient absorption and accumulation capabilities, leading to a gradual increase in TP and TN in the soil (Spohn et al., 2016). However, regression analysis showed that elevated TP content might cause certain plant species to overgrow, inducing changes in ecological niches and establishing competitive advantages in the ecosystem, consequently reducing community diversity and stability (Liu et al., 2022).

In the initial stages of post-fire succession, the soil pH turned slightly alkaline, owing to the hydrolysis of alkaline cation oxides present in the ash resulting from the fire (Ballard, 2000; Korb et al., 2004). As the recovery time progressed, in the 5 to 15-year stage, the pH significantly decreased. Likely due to enhanced root respiration, increased release of organic acids from plant roots, and microbial decomposition of dead plant materials (Lu et al., 2007). Although pH has a significant impact on species diversity and phylogenetic diversity (**Figure 7**), it does not have an appreciable effect on community stability (**Figure 6B**). The relationship between biodiversity and ecosystem stability is complex, and different species may exhibit varying degrees of tolerance and adaptability to soil pH. Some species may be more sensitive to pH changes, while others may have greater adaptability, enabling them to survive under different pH conditions. Therefore, species with higher adaptability may contribute to maintaining the stability of the community (Liu et al., 2022).

The burnt soil exhibited higher levels of AK and AP compared to the unburnt, which gradually decreased with increased recovery time, consistent with other research findings (Snyman, 2015; Hinojosa et al., 2019; Raiesi and Pejman, 2021). Plant communities at different successional stages after wildfire have different nutrient requirements and cycling patterns (Zhou et al., 2022b). The wildfire may lead to the combustion of organic matter and plant residues in the soil, releasing nutrients previously absorbed and stored in plant tissues during growth and metabolism, resulting in relatively higher levels of AP and AK in the soil (Moya et al., 2019). The correlation between AP and AK content and community stability may be attributed to the enhanced capacity of plant roots to absorb AP and AK, leading to their gradual reduction (**Figure 6**). This filtration process reduces plant species with lower adaptability and weaker competitiveness (Teixeira et al., 2022), while promoting an increase in plant species with stronger adaptability and broader ecological niches, thereby maintaining ecosystem diversity and stability (Jiang et al., 2022).

In the early stages of post-fire succession, the content of NO_3_–N and NH_4_^+^-N was not significantly lower than in unburnt areas, and with the progression of succession, a trend of initial decrease followed by an increase was observed. This trend could be linked to the combustion of vegetation and organic matter during the fire, releasing nutrients, including NO_3_–N and NH_4_^+^–N, into the soil. Initial nutrient mineralization may lead to an increase in nitrogen content in the soil in the short term after the fire (Korb et al., 2004). As succession proceeds, changes in vegetation types with different nitrogen absorption and release characteristics may contribute to the dynamic changes in NO_3_–N and NH_4_^+^–N (Li et al., 2023c).

The SOC content was significantly lower in the burnt areas immediately after the fire, but gradually increased over time. This could be ascribed to the gradual accumulation of contributions from plants and other organic matter as vegetation was re-established and succession occurred (Li et al., 2023b). New inputs of organic matter may include root secretions, litter, and dead plants and microbes, contributing to the gradual increase in SOC (Zhu et al., 2023).

The feedback mechanisms between soil and vegetation are dynamic and can continuously improve soil conditions, providing suitable habitats for various plants (Yan et al., 2022). Therefore, as the recovery period increases, positive succession occurs in the plant community, leading to a continuous enhancement of community stability (Zhang et al., 2022).

### 4.3 Restoration mechanisms of community structure and soil properties

PLS-PM serves as an effective tool for understanding the interactions between post-fire community succession and various influencing factors in a complex yet straightforward manner. The results of the PLS-PM model showed (**Figure 8**) that with the increase of restoration time, soil properties had a direct effect on community stability that was gradually decreasing, while the indirect effect was gradually increasing, and the total effect of the whole successional process was always positive. This may imply that the improvement of soil properties is beneficial to the development and adaptation of the community to some extent. However, when soil properties reach a certain level, it may lead to over competition and imbalance within the community, thus reducing community stability (Yan et al., 2022). Soil nutrient increase within a certain range can enhance community diversity, but beyond this level, its contribution to community diversity may gradually diminish, becoming a limiting factor (Galindo et al., 2022). The dynamic changes in indirect effects are attributed to the enhancement of soil properties promoting community species diversity, and reinforcing community stability. This phenomenon may be related to the diversity-stability hypothesis, suggesting that communities with higher species diversity are more resistant and recover better from biotic and abiotic disturbances, maintaining community structure and functionality (Liang et al., 2022; Zhao et al., 2022). Overall, the impact of soil properties on community stability depends not only on the changes in soil properties but also on their interactions with other ecological factors, varying over time and space.

Both direct and indirect effects of phylogenetic diversity on community stability diminished. Throughout the restoration process, its direct effect shifted from a positive effect in the early stages to a negative effect in the middle and late stages, but the indirect effect was always positive. This may be attributed to two main reasons: firstly, the convergence of species’ functional traits and ecological niches as succession progresses, resulting from competition and selection leading to trait convergence and filtering (Gross et al., 2014). Consequently, the influence of phylogenetic diversity on community stability gradually weakens. Secondly, changes in species asynchrony and dominance (Wang and Loreau, 2016). Species asynchrony refers to species responding to environmental changes asynchronously, reducing community variability (Jiang et al., 2022). Species dominance refers to the relative importance of species in the community, influencing the average performance of the community (Grman et al., 2010). As community succession progresses, species asynchrony and dominance may change due to interactions among species and environmental changes, leading to an increase in species synchrony and uniformity (Zhang et al., 2018). Consequently, the impact of phylogenetic diversity on community stability undergoes a positive-to-negative transition.

Species diversity shows a consistently positive direct effect on community stability at different stages, which is useful in strengthening as the community vegetation recovers. This phenomenon may be related to the diversity-stability hypothesis (Yan et al., 2022), where complementary, redundant, buffering, and insurance effects among species lead to a more stable species composition and functional allocation within the community (Zhao et al., 2022). Our model effectively highlights species diversity as the most direct influencing factor on community stability during the process of community vegetation succession.

Community stability refers to the ability to maintain relative equilibrium and return to a stable state in the face of external disturbances. It is an important indicator of post-fire community resilience (Wagg et al., 2022). The results of this study indicate that, during succession, the post-fire landscape enhances community stability by continually adjusting community composition and coordinating interactions between species diversity and phylogenetic diversity (**Figure 8**). Observing the entire model’s succession process and trends, the community gradually transitions toward an unburnt state.

From the results of the PLS-PM model (**Figure 8**), it is evident that different factors dominate the influence on community stability at different stages of succession. In the initial stage, soil properties play a predominant role. As succession enters the middle to later stages, the direct impact of soil on stability gradually weakens. In the 23a stage, the soil exhibited a negative impact on community stability, while species diversity occurred. In the early stages, soil factors are key determinants of stability, as soil nutrients, moisture, and texture may directly affect plant growth and niche distribution (Tian et al., 2023). As succession progresses, the positive impact of species diversity on community stability mainly arises from the invasion and competition of different species. The decline in phylogenetic diversity may indicate that, over an extended period, species became more phylogenetically similar or that some species were replaced or disappeared, resulting in a decrease in phylogenetic diversity (Ritchie et al., 2021). This shift may be driven by competition or dispersion (Webb et al., 2002). In summary, at different stages of post-fire succession, various ecological factors and processes have distinct effects on community stability. These relationships are dynamic and subject to change over time.

## 5 Conclusion

In this study on post-fire vegetation communities at the northeastern margin of the Qinghai-Tibet Plateau, we utilized PLS-PM model to assess the resilience of ecosystems to severe fire disturbances and explored the complex interactions between plant community succession and soil properties following wildfires. Our findings demonstrate that species diversity initially drives community recovery, with pioneer species rapidly establishing dominance due to their adaptive traits. As succession progresses, the role of competitive exclusion becomes more pronounced, leading to higher community stability and uniform distribution of species. Phylogenetic diversity, assessed through MPD and MNTD indices, reveals that evolutionary relatedness among species contributes significantly to community resilience and functionality. Our innovative approach of integrating phylogenetic diversity into ecological studies provides a deeper understanding of post-fire recovery mechanisms, highlighting the importance of both deterministic and stochastic processes in shaping community structure. Additionally, the dynamic changes in soil properties, such as TP and pH, underscore their critical role in influencing nutrient cycling and community stability. Overall, our research offers novel insights into the restoration mechanisms of plant communities, emphasizing the synergistic effects of species diversity, phylogenetic diversity, and soil properties on ecosystem resilience and stability post-fire.

## Data availability statement

The original contributions presented in the study are included in the article/**Supplementary Material**, further inquiries can be directed to the corresponding author/s.

## Author contributions

ZL: Conceptualization, Data curation, Formal analysis, Methodology, Software, Visualization, Writing – original draft. JW: Conceptualization, Formal analysis, Methodology, Writing – original draft. XZ: Conceptualization, Data curation, Funding acquisition, Project administration, Supervision, Validation, Writing – review & editing. QT: Conceptualization, Funding acquisition, Methodology, Project administration, Supervision, Validation, Writing – review & editing. WH: Data curation, Investigation, Writing – review & editing. XC: Investigation, Writing – review & editing.
