# Supplementary material for: Dynamic restoration mechanism of plant community in the burned area of northeastern margin of Qinghai-Tibet Plateau
Source: Front Plant Sci. 2024 Jul 25;15:1368814. doi: 10.3389/fpls.2024.1368814 (PMC11306190; doi:10.3389/fpls.2024.1368814)
Supplement: Supplementary file 1 [file DataSheet_1.docx]

Supplementary Material

# Supplementary Figures and Tables

#
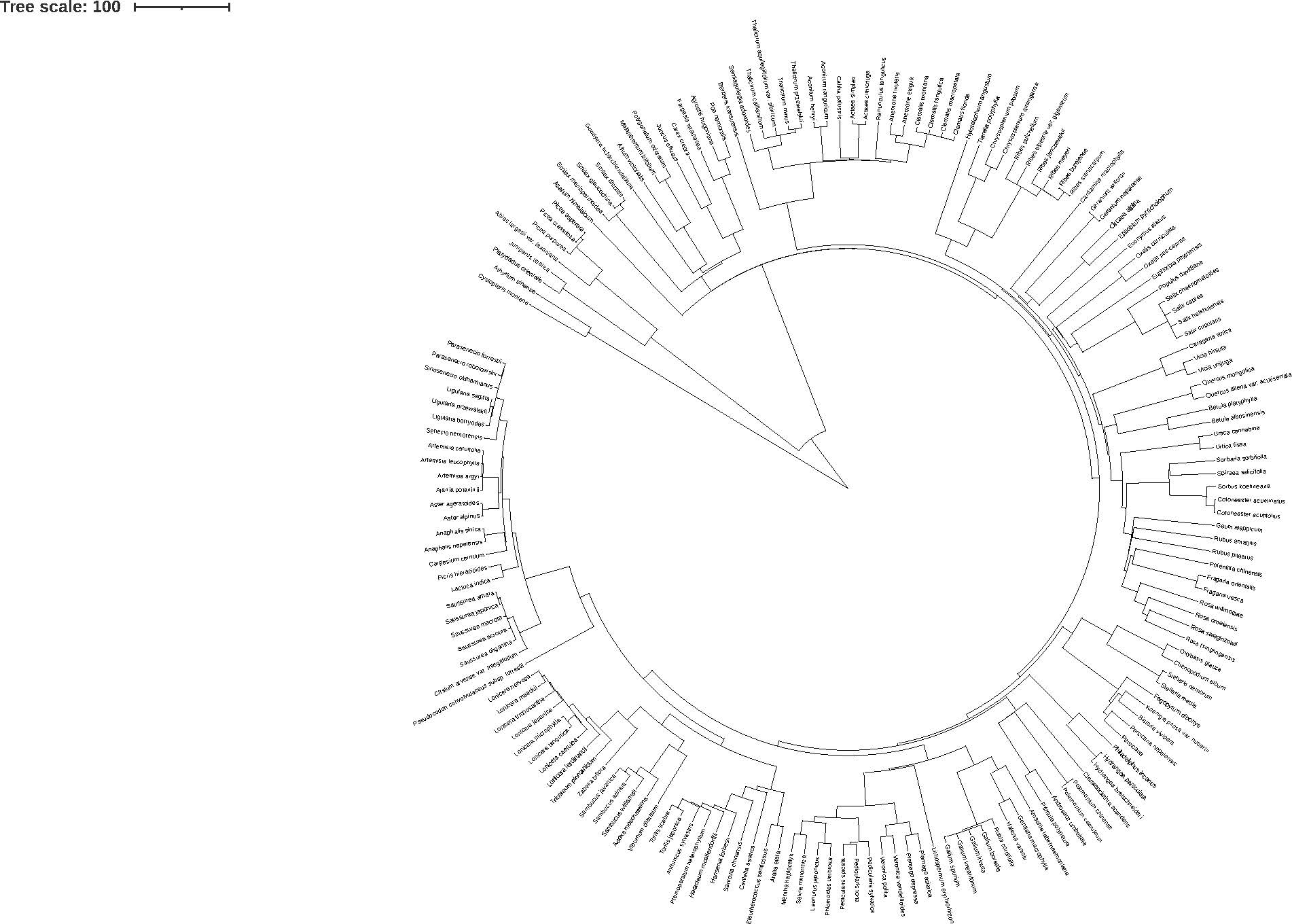


# Fig. S1. The Tree of plants.


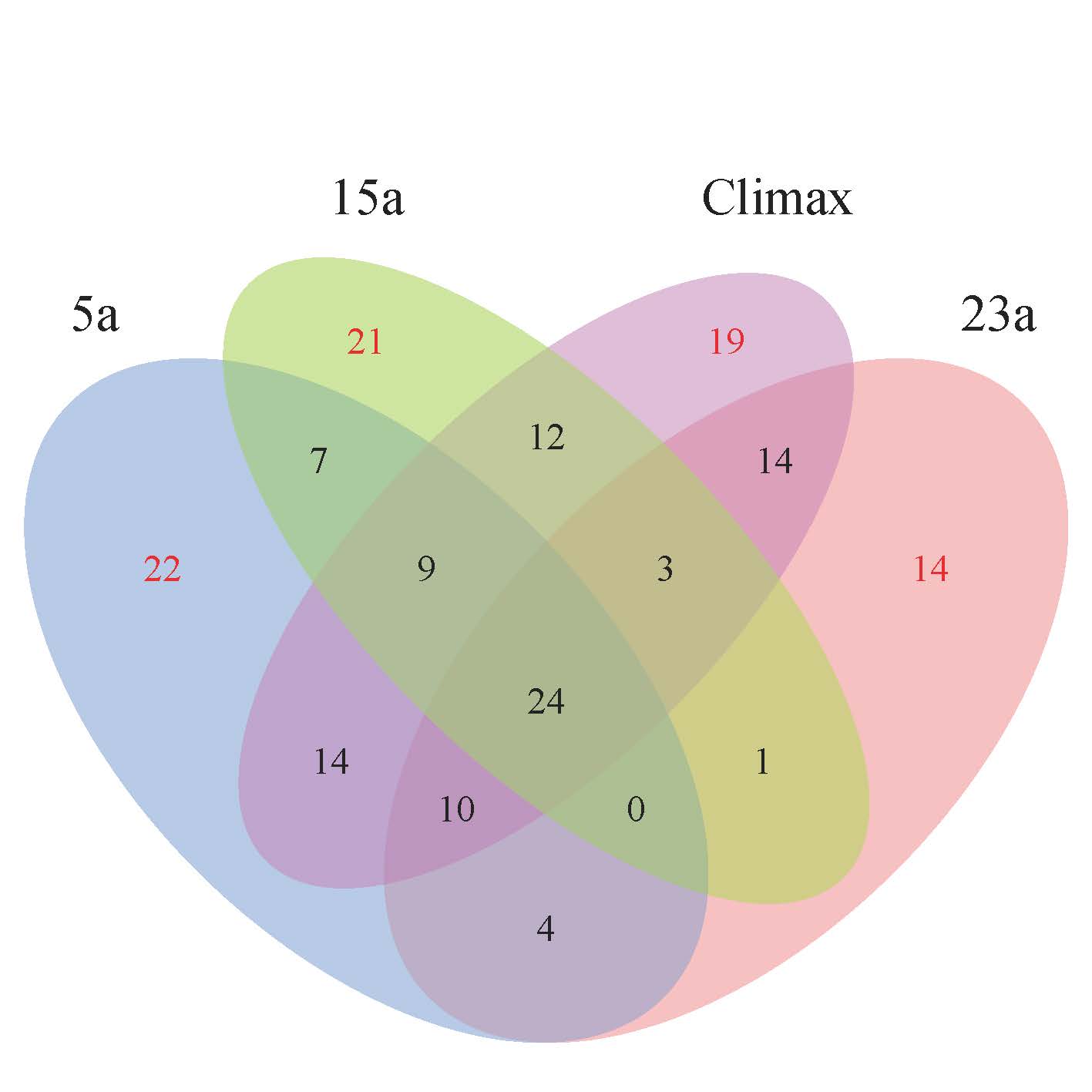


**Fig. S2.** Venn diagram of the different stages of vegetation restoration. Each ellipse represents a species group in a stage. External numbers indicate restoration years. Red font showed stage-specific endemic plant totals. Black numbers within ellipses indicated species shared among stages.

**Table S1.** Basic situation of communities and the sampling sites at different succession stages

| Succession stages | Forest station | Year of forest fire | Fire intensity | Management and protection measures | Slope (°) | Altitude (m) | Soil type | Depth of soil layer (cm) | Composition of community |
| --- | --- | --- | --- | --- | --- | --- | --- | --- | --- |
| 5a | Dala | 2016 | High fire intensity | Closure | 30~44 | 2581~2870 | Brown soil | 40~200 | 90 species 66 genus in 34 families |
| 15a | Yiwa | 2005 | High fire intensity | Closure | 22~52 | 2981~ 3390 | Gray brown soil | 49~107 | 77 species 59 genus in 31 families |
| 23a | Nagai | 1997 | High fire intensity | Closure | 27~ 38 | 2680~ 3100 | Gray brown soil | 52~210 | 70 species 48 genus in 31 families |
| Climax community | Yiwa, Dala and Nagai | No | No | Closure | 21~ 54 | 2575~ 3410 | Brown soil，gray brown soil | 36~308 | 105 species 72 genus in 38 families |

**Table S2.** List of plants in the study area

| SPECIES | GENUS | FAMILY |
| --- | --- | --- |
| *Ligularia botryodes* | Ligularia | Asteraceae |
| *Ligularia sagitta* | Ligularia | Asteraceae |
| *Ligularia przewalskii* | Ligularia | Asteraceae |
| *Anaphalis sinica* | Anaphalis | Asteraceae |
| *Anaphalis nepalensis* | Anaphalis | Asteraceae |
| *Aster alpinus* | Aster | Asteraceae |
| *Aster ageratoides* | Aster | Asteraceae |
| *Sinosenecio oldhamianus* | Sinosenecio | Asteraceae |
| *Saussurea oligantha* | Saussurea | Asteraceae |
| *Saussurea amara* | Saussurea | Asteraceae |
| *Saussurea acroura* | Saussurea | Asteraceae |
| *Saussurea japonica* | Saussurea | Asteraceae |
| *Cirsium arvense* var*. integrifolium* | Cirsium | Asteraceae |
| *Parasenecio forrestii* | Parasenecio | Asteraceae |
| *Parasenecio roborowskii* | Parasenecio | Asteraceae |
| *Senecio nemorensis* | Senecio | Asteraceae |
| *Saussurea macrota* | Saussurea | Asteraceae |
| *Carpesium cernuum* | Carpesium | Asteraceae |
| *Lactuca indica* | Lactuca | Asteraceae |
| *Artemisia leucophylla* | Artemisia | Asteraceae |
| *Artemisia caruifolia* | Artemisia | Asteraceae |
| *Ajania potaninii* | Ajania | Asteraceae |
| *Artemisia argyi* | Artemisia | Asteraceae |
| *Picris hieracioides* | Picris | Asteraceae |
| *Cystopteris montana* | Cystopteris | Cystopteridaceae |
| *Athyrium sinense* | Athyrium | Athyriaceae |
| *Carex crebra* | Carex | Cyperaceae |
| *Thalictrum przewalskii* | Thalictrum | Ranunculaceae |
| *Clematis florida* | Clematis | Ranunculaceae |
| *Ranunculus tanguticus* | Ranunculus | Ranunculaceae |
| *Semiaquilegia adoxoides* | Semiaquilegia | Ranunculaceae |
| *Clematis macropetala* | Clematis | Ranunculaceae |
| *Clematis montana* | Clematis | Ranunculaceae |
| *Thalictrum callianthum* | Thalictrum | Ranunculaceae |
| *Caltha palustris* | Caltha | Ranunculaceae |
| *Actaea cimicifuga* | Actaea | Ranunculaceae |
| *Clematis tangutica* | Clematis | Ranunculaceae |
| *Thalictrum minus* | Thalictrum | Ranunculaceae |
| *Anemone rivularis* | Anemone | Ranunculaceae |
| *Thalictrum aquilegiifolium* var*. sibiricum* | Thalictrum | Ranunculaceae |
| *Aconitum tanguticum* | Aconitum | Ranunculaceae |
| *Aconitum henryi* | Aconitum | Ranunculaceae |
| *Actaea simplex* | Actaea | Ranunculaceae |
| *Anemone exigua* | Anemone | Ranunculaceae |
| *Rosa omeiensis* | Rosa | Rosaceae |
| *Rosa willmottiae* | Rosa | Rosaceae |
| *Rosa sweginzowii* | Rosa | Rosaceae |
| *Fragaria orientalis* | Fragaria | Rosaceae |
| *Rosa tsinglingensis* | Rosa | Rosaceae |
| *Fragaria vesca* | Fragaria | Rosaceae |
| *Cotoneaster acutifolius* | Cotoneaster | Rosaceae |
| *Geum aleppicum* | Geum | Rosaceae |
| *Rubus amabilis* | Rubus | Rosaceae |
| *Potentilla chinensis* | Potentilla | Rosaceae |
| *Spiraea salicifolia* | Spiraea | Rosaceae |
| *Sorbaria sorbifolia* | Sorbaria | Rosaceae |
| *Sorbus koehneana* | Sorbus | Rosaceae |
| *Cotoneaster acuminatus* | Cotoneaster | Rosaceae |
| *Rubus pileatus* | Rubus | Rosaceae |
| *Gentiana macrophylla* | Gentiana | Gentianaceae |
| *Halenia vaniotii* | Halenia | Gentianaceae |
| *Epilobium pyrricholophum* | Epilobium | Onagraceae |
| *Circaea alpina* | Circaea | Onagraceae |
| *Amsonia tabernaemontana* | Amsonia | Apocynaceae |
| *Caragana sinica* | Caragana | Fabaceae |
| *Vicia hirsuta* | Vicia | Fabaceae |
| *Vicia unijuga* | Vicia | Fabaceae |
| *Polygonatum odoratum* | Polygonatum | Asparagaceae |
| *Maianthemum bifolium* | Maianthemum | Asparagaceae |
| *Plantago asiatica* | Plantago | Plantaginaceae |
| *Veronica polita* | Veronica | Plantaginaceae |
| *Plantago depressa* | Plantago | Plantaginaceae |
| *Veronica vandellioides* | Veronica | Plantaginaceae |
| *Pedicularis torta* | Pedicularis | Orobanchaceae |
| *Pedicularis sylvatica* | Pedicularis | Orobanchaceae |
| *Pedicularis spicata* | Pedicularis | Orobanchaceae |
| *Galium boreale* | Galium | Rubiaceae |
| *Galium kinuta* | Galium | Rubiaceae |
| *Galium linearifolium* | Galium | Rubiaceae |
| *Rubia cordifolia* | Rubia | Rubiaceae |
| *Galium spurium* | Galium | Rubiaceae |
| *Polemonium caeruleum* | Polemonium | Polemoniaceae |
| *Polemonium chinense* | Polemonium | Polemoniaceae |
| *Geranium wilfordii* | Geranium | Geraniaceae |
| *Geranium nepalense* | Geranium | Geraniaceae |
| *Sanicula chinensis* | Sanicula | Apiaceae |
| *Pternopetalum heterophyllum* | Pternopetalum | Apiaceae |
| *Centella asiatica* | Centella | Apiaceae |
| *Torilis scabra* | Torilis | Apiaceae |
| *Heracleum moellendorffii* | Heracleum | Apiaceae |
| *Hansenia forbesii* | Hansenia | Apiaceae |
| *Torilis japonica* | Torilis | Apiaceae |
| *Anthriscus sylvestris* | Anthriscus | Apiaceae |
| *Urtica cannabina* | Urtica | Urticaceae |
| *Urtica fissa* | Urtica | Urticaceae |
| *Cardamine macrophylla* | Cardamine | Brassicaceae |
| *Chrysosplenium qinlingense* | Chrysosplenium | Saxifragaceae |
| *Chrysosplenium pilosum* | Chrysosplenium | Saxifragaceae |
| *Tiarella polyphylla* | Tiarella | Saxifragaceae |
| *Chenopodium album* | Chenopodium | Amaranthaceae |
| *Oxybasis glauca* | Oxybasis | Amaranthaceae |
| *Mentha haplocalyx* | Mentha | Lamiaceae |
| *Leonurus japonicus* | Leonurus | Lamiaceae |
| *Phlomoides umbrosa* | Phlomoides | Lamiaceae |
| *Salvia miltiorrhiza* | Salvia | Lamiaceae |
| *Primula polyneura* | Primula | Primulaceae |
| *Androsace umbellata* | Androsace | Primulaceae |
| *Fagopyrum dibotrys* | Fagopyrum | Polygonaceae |
| [*Persicaria lapathifolia*](http://www.iplant.cn/info/Persicaria%20lapathifolia) | Persicaria | Polygonaceae |
| *Koenigia pilosa* var*. hubertii* | Koenigia | Polygonaceae |
| *Bistorta vivipara* | Bistorta | Polygonaceae |
| *Persicaria nepalensis* | Persicaria | Polygonaceae |
| *Lonicera microphylla* | Lonicera | Caprifoliaceae |
| *Lonicera maackii* | Lonicera | Caprifoliaceae |
| *Lonicera trichosantha* | Lonicera | Caprifoliaceae |
| *Triosteum pinnatifidum* | Triosteum | Caprifoliaceae |
| *Lonicera caerulea* | Lonicera | Caprifoliaceae |
| *Lonicera ferdinandi* | Lonicera | Caprifoliaceae |
| *Lonicera nervosa* | Lonicera | Caprifoliaceae |
| *Lonicera japonica* | Lonicera | Caprifoliaceae |
| *Zabelia biflora* | Zabelia | Caprifoliaceae |
| *Lonicera tangutica* | Lonicera | Caprifoliaceae |
| *Lithospermum erythrorhizon* | Lithospermum | Boraginaceae |
| *Goodyera schlechtendaliana* | Goodyera | Orchidaceae |
| *Pseudocodon convolvulaceus* subsp*. forrestii* | Pseudocodon | Campanulaceae |
| *Allium victorialis* | Allium | Amaryllidaceae |
| *Asarum himalaicum* | Asarum | Aristolochiaceae |
| *Sambucus javanica* | Sambucus | Adoxaceae |
| *Viburnum dilatatum* | Viburnum | Adoxaceae |
| *Adoxa moschatellina* | Adoxa | Adoxaceae |
| *Sambucus williamsii* | Sambucus | Adoxaceae |
| *Sambucus adnata* | Sambucus | Adoxaceae |
| *Oxalis corniculata* | Oxalis | Oxalidaceae |
| *Oxalis pes caprae* | Oxalis | Oxalidaceae |
| *Euphorbia pekinensis* | Euphorbia | Euphorbiaceae |
| *Stellaria nemorum* | Stellaria | Caryophyllaceae |
| *Stellaria media* | Stellaria | Caryophyllaceae |
| *Poa nemoralis* | Poa | Poaceae |
| *Fargesia spathacea* | Fargesia | Poaceae |
| *Agrostis hugoniana* | Agrostis | Poaceae |
| *Juncus effusus* | Juncus | Juncaceae |
| *Hylotelephium angustum* | Hylotelephium | Crassulaceae |
| *Eleutherococcus senticosus* | Eleutherococcus | Araliaceae |
| *Aralia elata* | Aralia | Araliaceae |
| *Salix chaenomeloides* | Salix | Salicaceae |
| *Salix caprea* | Salix | Salicaceae |
| *Salix heishuiensis* | Salix | Salicaceae |
| *Salix cupularis* | Salix | Salicaceae |
| *Populus davidiana* | Populus | Salicaceae |
| *Philadelphus incanus* | Philadelphus | Hydrangeaceae |
| *Hydrangea bretschneideri* | Hydrangea | Hydrangeaceae |
| *Hydrangea paniculata* | Hydrangea | Hydrangeaceae |
| *Ribes burejense* | Ribes | Grossulariaceae |
| *Ribes stenocarpum* | Ribes | Grossulariaceae |
| *Ribes meyeri* | Ribes | Grossulariaceae |
| *Ribes janczewskii* | Ribes | Grossulariaceae |
| *Ribes pulchellum* | Ribes | Grossulariaceae |
| *Ribes alpestre* var*. giganteum* | Ribes | Grossulariaceae |
| *Smilax glaucochina* | Smilax | Smilacaceae |
| *Smilax menispermoidea* | Smilax | Smilacaceae |
| *Smilax discotis* | Smilax | Smilacaceae |
| *Clematoclethra scandens* | Clematoclethra | Actinidiaceae |
| *Berberis kansuensis* | Berberis | Berberidaceae |
| *Euonymus alatus* | Euonymus | Celastraceae |
| *Betula albosinensis* | Betula | Betulaceae |
| *Betula platyphylla* | Betula | Betulaceae |
| *Quercus mongolica* | Quercus | Fagaceae |
| *Quercus aliena* var*. acutiserrata* | Quercus | Fagaceae |
| *Picea crassifolia* | Picea | Pinaceae |
| *Picea asperata* | Picea | Pinaceae |
| *Picea purpurea* | Picea | Pinaceae |
| *Abies fargesii* var*. faxoniana* | Abies | Pinaceae |
| *Juniperus tibetica* | Juniperus | Cupressaceae |
| *Platycladus orientalis* | Platycladus | Cupressaceae |

**Table S3.** Venn Overlap of various restoration years.

| 5a | 5a-15a | 5a-15a-ck | 5a-23a | 5a+23a+15a+ck | 5a+23a+ck | 5a+ck | 15a | 15a+ck | 23a | 15a+23a | 23a+15a+ck | 23a+ck | ck |
| --- | --- | --- | --- | --- | --- | --- | --- | --- | --- | --- | --- | --- | --- |
| Ajania potaninii | Artemisia caruifolia | Betula platyphylla | Actaea simplex | Actaea cimicifuga | Artemisia leucophylla | Amsonia tabernaemontana | Agrostis hugoniana | Abies fargesii var. faxoniana | Aconitum henryi | Polemonium chinense | Polygonatum odoratum | Asarum himalaicum | Aconitum tanguticum |
| Caltha palustris | Carpesium cernuum | Cotoneaster acutifolius | Polemonium caeruleum | Anaphalis sinica | Chrysosplenium qinlingense | Anthriscus sylvestris | Athyrium sinense | Anemone exigua | Anaphalis nepalensis |  | Pseudocodon convolvulaceus subsp. forrestii | Galium linearifolium | Adoxa moschatellina |
| Cirsium arvense var. integrifolium | Clematoclethra scandens | Geum aleppicum | Salix cupularis | Aster ageratoides | Circaea alpina | Aralia elata | Cotoneaster acuminatus | Bistorta vivipara | Chenopodium album |  | Ribes janczewskii | Lithospermum erythrorhizon | Allium victorialis |
| Clematis montana | Picris hieracioides | Halenia vaniotii | Senecio nemorensis | Berberis kansuensis | Eleutherococcus senticosus | Artemisia argyi | Euphorbia pekinensis | Geranium wilfordii | Euonymus alatus |  |  | Lonicera ferdinandi | Androsace umbellata |
| Epilobium pyrricholophum | Ribes alpestre var. giganteum | Lonicera nervosa |  | Betula albosinensis | Fragaria orientalis | Cardamine macrophylla | Gentiana macrophylla | Heracleum moellendorffii | Fagopyrum dibotrys |  |  | Oxalis corniculata | Anemone rivularis |
| Hydrangea paniculata | Salix heishuiensis | Picea asperata |  | Carex crebra | Galium kinuta | Clematis macropetala | Hansenia forbesii | Lonicera japonica | Galium boreale |  |  | Plantago depressa | Aster alpinus |
| Lactuca indica | Saussurea macrota | Plantago asiatica |  | Clematis florida | Saussurea japonica | Geranium nepalense | Hylotelephium angustum | Persicaria | Ligularia sagitta |  |  | Potentilla chinensis | Caragana sinica |
| Maianthemum bifolium |  | Saussurea oligantha |  | Cystopteris montana | Thalictrum aquilegiifolium var. sibiricum | Hydrangea bretschneideri | Juncus effusus | Pternopetalum heterophyllum | Lonicera maackii |  |  | Primula polyneura | Centella asiatica |
| Oxybasis glauca |  | Veronica polita |  | Fargesia spathacea | Urtica fissa | Lonicera microphylla | Koenigia pilosa var. hubertii | Rosa omeiensis | Picea crassifolia |  |  | Ribes burejense | Chrysosplenium pilosum |
| Pedicularis spicata |  |  |  | Fragaria vesca | Vicia unijuga | Mentha haplocalyx | Leonurus japonicus | Rosa tsinglingensis | Poa nemoralis |  |  | Ribes meyeri | Clematis tangutica |
| Pedicularis sylvatica |  |  |  | Galium spurium |  | Quercus mongolica | Parasenecio forrestii | Salvia miltiorrhiza | Salix caprea |  |  | Ribes pulchellum | Goodyera schlechtendaliana |
| Pedicularis torta |  |  |  | Ligularia botryodes |  | Smilax glaucochina | Parasenecio roborowskii | Zabelia biflora | Salix chaenomeloides |  |  | Ribes stenocarpum | Juniperus tibetica |
| Populus davidiana |  |  |  | Ligularia przewalskii |  | Sorbaria sorbifolia | Persicaria nepalensis |  | Thalictrum minus |  |  | Sorbus koehneana | Oxalis pes-caprae |
| Quercus aliena var. acutiserrata |  |  |  | Lonicera caerulea |  | Tiarella polyphylla | Ranunculus tanguticus |  | Triosteum pinnatifidum |  |  | Veronica vandellioides | Phlomoides umbrosa |
| Sambucus williamsii |  |  |  | Lonicera tangutica |  |  | Sambucus adnata |  |  |  |  |  | Picea purpurea |
| Saussurea amara |  |  |  | Lonicera trichosantha |  |  | Sambucus javanica |  |  |  |  |  | Platycladus orientalis |
| Sinosenecio oldhamianus |  |  |  | Philadelphus incanus |  |  | Smilax menispermoidea |  |  |  |  |  | Saussurea acroura |
| Smilax discotis |  |  |  | Rosa sweginzowii |  |  | Stellaria media |  |  |  |  |  | Semiaquilegia adoxoides |
| Spiraea salicifolia |  |  |  | Rosa willmottiae |  |  | Stellaria nemorum |  |  |  |  |  | Thalictrum przewalskii |
| Urtica cannabina |  |  |  | Rubia cordifolia |  |  | Thalictrum callianthum |  |  |  |  |  |  |
| Viburnum dilatatum |  |  |  | Rubus amabilis |  |  | Torilis japonica |  |  |  |  |  |  |
| Vicia hirsuta |  |  |  | Rubus pileatus |  |  |  |  |  |  |  |  |  |
|  |  |  |  | Sanicula chinensis |  |  |  |  |  |  |  |  |  |
|  |  |  |  | Torilis scabra |  |  |  |  |  |  |  |  |  |

**Table S4.** The relative abundance at the genu-level of plant communities with different recovery years.

| Taxonomy | 5a | 15a | 23a | ck |
| --- | --- | --- | --- | --- |
| Carex | 0.161643 | 0.093117 | 0.183949 | 0.213423 |
| Rubus | 0.153274 | 0.003686 | 0.089219 | 0.244667 |
| Cystopteris | 0.14098 | 0.005682 | 0.069783 | 0.042653 |
| Veronica | 0.128159 | 0.083357 | 0 | 0 |
| Fargesia | 0.015194 | 0.014584 | 0.071307 | 0.185369 |
| Rubus.1 | 0.083548 | 0.002949 | 0.002162 | 0.039997 |
| Fragaria | 0.007694 | 0.132457 | 0.000439 | 0 |
| Circaea | 0.015014 | 0 | 0.016292 | 0.080722 |
| Ligularia | 0.012814 | 0.035026 | 0.013352 | 0.006037 |
| Betula.1 | 0.032866 | 0.018737 | 0.001441 | 0 |
| Oxalis | 0 | 0 | 0.029217 | 0.090432 |
| Galium.1 | 0.015855 | 0.012251 | 0.018798 | 0.037044 |
| Plantago | 0.029839 | 0.011533 | 0 |  |
| Athyrium | 0 | 0.045337 | 0 |  |
| Other | 0.203119 | 0.541284 | 0.504041 |  |

**Table S5**. Effects of the restoration time (Year) with taxonomy diversity and phylogentic diversity.

| Factors | years | |
| --- | --- | --- |
|  | F | P |
| Margalef | 4.834 | 0.0103 |
| Pielou | 2.547 | 0.0834 |
| Simpson | 1.112 | 0.366 |
| Shannon | 0.549 | 0.654 |
| PD | 10.01 | 0.000267 |
| MPD | 3.74 | 0.0268 |
| MNTD | 1.894 | 0.162 |
| NRI | 3.833 | 0.0247 |
| NTI | 2.357 | 0.101 |

**Table S6** Effects of the restoration time (Year) and soil depth on the soil organic carbon (SOC), total nitrogen (TN), total phosphorus (TP), total potassium (TK), available phosphorus (AP), available potassium (AK), ammonium nitrogen (NH_4_-N), nitrate nitrogen (NO_3_-N) and soil pH value.

| Factors | Time | | Depth | | Time×Depth | |
| --- | --- | --- | --- | --- | --- | --- |
|  | F | P | F | P | F | P |
| SOC | 16.354 | 5.71E-08 | 20.013 | 1.87E-07 | 1.265 | 2.86E-01 |
| TN | 15.845 | 8.73E-08 | 5.852 | 5.00E-03 | 0.73 | 6.27E-01 |
| pH | 24.322 | 1.42E-10 | 1.29 | 2.82E-01 | 0.534 | 7.80E-01 |
| TK | 16.686 | 4.35E-08 | 1.474 | 2.37E-01 | 0.137 | 9.91E-01 |
| TP | 57.401 | 5.25E-18 | 1.754 | 1.81E-01 | 0.176 | 9.83E-01 |
| AP | 8.758 | 6.14E-05 | 7.452 | 1.00E-03 | 0.388 | 8.84E-01 |
| AK | 6.391 | 0.000753 | 10.045 | 0.000164 | 1.987 | 0.081 |
| NH_4_-N | 25.422 | 6.75E-11 | 6.678 | 2.00E-03 | 0.693 | 6.56E-01 |
| NO_3_-N | 8.682 | 6.63E-05 | 10.355 | 1.29E-04 | 0.136 | 9.91E-01 |

**Table S7** The results of the Mantel test

| Plants diversity | Environment | r | p.value | rd | pd |
| --- | --- | --- | --- | --- | --- |
| Species_diversity | TN | -0.08385 | 0.912 | < 0.1 | >= 0.05 |
| Species_diversity | TP | 0.216781 | 0.014 | >= 0.2 | 0.01 - 0.05 |
| Species_diversity | TK | 0.028479 | 0.295 | < 0.1 | >= 0.05 |
| Species_diversity | AP | 0.036099 | 0.347 | < 0.1 | >= 0.05 |
| Species_diversity | AK | 0.073008 | 0.2 | < 0.1 | >= 0.05 |
| Species_diversity | NH_4_^+^-N | -0.05396 | 0.811 | < 0.1 | >= 0.05 |
| Species_diversity | NO_3_^-^-N | -0.05769 | 0.739 | < 0.1 | >= 0.05 |
| Species_diversity | pH | 0.332135 | 0.001 | >= 0.2 | < 0.01 |
| Species_diversity | SOC | 0.160128 | 0.049 | 0.1 - 0.2 | 0.01 - 0.05 |
| Phylogenetic_diversity | TN | -0.01584 | 0.565 | < 0.1 | >= 0.05 |
| Phylogenetic_diversity | TP | 0.282904 | 0.006 | >= 0.2 | < 0.01 |
| Phylogenetic_diversity | TK | 0.152296 | 0.033 | 0.1 - 0.2 | 0.01 - 0.05 |
| Phylogenetic_diversity | AP | 0.154704 | 0.073 | 0.1 - 0.2 | >= 0.05 |
| Phylogenetic_diversity | AK | 0.122501 | 0.108 | 0.1 - 0.2 | >= 0.05 |
| Phylogenetic_diversity | NH_4_^+^-N | 0.00473 | 0.436 | < 0.1 | >= 0.05 |
| Phylogenetic_diversity | NO_3_^-^-N | 9.31E-06 | 0.459 | < 0.1 | >= 0.05 |
| Phylogenetic_diversity | pH | 0.317211 | 0.006 | >= 0.2 | < 0.01 |
| Phylogenetic_diversity | SOC | 0.169581 | 0.064 | 0.1 - 0.2 | >= 0.05 |
